# Full and simplified assessment of left ventricular diastolic function in covid‐19 patients admitted to ICU: Feasibility, incidence, and association with mortality

**DOI:** 10.1111/echo.15462

**Published:** 2022-10-06

**Authors:** Luigi La Via, Veronica Dezio, Cristina Santonocito, Marinella Astuto, Andrea Morelli, Stephen Huang, Antoine Vieillard‐Baron, Filippo Sanfilippo

**Affiliations:** ^1^ Department of Anaesthesia and Intensive Care “Policlinico‐San Marco” University Hospital Catania Italy; ^2^ Department Clinical Internal, Anesthesiological and Cardiovascular Sciences, University of Rome “La Sapienza”, Policlinico Umberto Primo Roma Italy; ^3^ Intensive Care Medicine, Nepean Hospital The University of Sydney Sydney Australia; ^4^ Service de Médecine Intensive Réanimation, Assistance Publique‐Hôpitaux de Paris University Hospital Ambroise Paré Boulogne‐Billancourt France

**Keywords:** e’, E/e’ ratio, guidelines, systolic function, tissue Doppler

## Abstract

**Purpose:**

Left ventricular diastolic dysfunction (LVDD) is associated with poor outcomes in the intensive care unit (ICU). Nonetheless, precise reporting of LVDD in COVID‐19 patients is currently lacking and assessment could be challenging.

**Methods:**

We performed an echocardiography study in COVID‐19 patients admitted to ICU with the aim to describe the feasibility of full or simplified LVDD assessment and its incidence. We also evaluated the association of LVDD or of single echocardiographic parameters with hospital mortality.

**Results:**

Between 06.10.2020 and 18.02.2021, full diastolic assessment was feasible in 74% (*n* = 26/35) of patients receiving a full echocardiogram study. LVDD incidence was 46% (*n* = 12/26), while the simplified assessment produced different results (incidence 81%, *n* = 21/26). Nine patients with normal function on full assessment had LVDD with simplified criteria (grade I = 2; grade II = 3; grade III = 4). Nine patients were hospital‐survivors (39%); the incidence of LVDD (full assessment) was not different between survivors (*n* = 2/9, 22%) and non‐survivors (*n* = 10/17, 59%; *p* = .11). The E/e’ ratio lateral was lower in survivors (7.4 [3.6] vs. non‐survivors 10.5 [6.3], *p* = .03). We also found that s’ wave was higher in survivors (average, *p* = .01).

**Conclusion:**

In a small single‐center study, assessment of LVDD according to the latest guidelines was feasible in three quarters of COVID‐19 patients. Non‐survivors showed a trend toward greater LVDD incidence; moreover, they had significantly worse s’ values (all) and higher E/e’ ratio (lateral).

## 1 INTRODUCTION

Coronavirus disease 19 (COVID‐19) pandemic has caused over 6 million deaths worldwide and these figures are likely underestimated.^1^ COVID‐19 infection may span from asymptomatic or mild and self‐limiting cases to severe illness requiring hospitalization where COVID‐19 may trigger a multi‐systemic infection involving different organs.^2^, ^3^, ^4^, ^5^ The lungs seem the most affected organ with possible development of interstitial pneumonia requiring hospitalization and intensive care unit (ICU) admission with mechanical ventilation in severe cases.^6^, ^7^, ^8^ A substantial cardiovascular impact in patients with COVID‐19 has been repeatedly demonstrated^9^; of note, even patients not requiring hospitalization have shown some degree of myocardial dysfunction with features of myocarditis on magnetic resonance imaging.^10^, ^11^, ^12^, ^13^

Severely ill COVID‐19 patients admitted to ICU may experience cardio‐circulatory failure and a fair amount of them may need support with catecholamine infusions. Different degrees of cardiac injury as evaluated by biomarkers^14^, ^15^, ^16^ or echocardiography^9^, ^17^ have been reported for COVID‐19 patients admitted to ICU. Several patterns of cardiovascular dysfunction have also been described: from signs of myocarditis or myocardial ischemia to significant hypovolemia (due to pyrexia and prolonged fasting), from right ventricular (RV) failure (influenced by mechanical ventilation and/or by micro/macro pulmonary embolism) to septic cardiovascular dysfunction due to super‐imposed bacterial or fungal infections.^10^, ^11^, ^13^, ^18^, ^19^ Moreover, a combination of these features could be coexistent in severely ill patients with COVID‐19. Interestingly, a gap of knowledge exists regarding the feasibility of precise characterization of left ventricular diastolic dysfunction (LVDD) according to the joint recommendations from the European Association of Cardiovascular Imaging (EACVI) and the American Society of Echocardiography (ASE)^20^ in this population of patients, which are at high risk for both chronic LVDD (i.e., history of hypertension and diabetes) or acute deterioration of their LV diastolic function. Of note, in non‐COVID‐19 critically ill patients, left ventricular diastolic dysfunction (LVDD) has received attention for its association with outcomes,^21^, ^22^, ^23^ while the same association has not been shown for left ventricular systolic dysfunction (LVSD).^24^ Echocardiography is crucial in diagnosing and grading LVDD and may help distinguish patterns of cardiovascular dysfunction, suggest therapeutic options, and track the changes with sequential monitoring.^25^

Our single‐center joined the international ECHO‐COVID study.^17^ With the purpose to fully characterize LVDD, we also collected tissue Doppler Imaging (TDI) and left atrial volume index (LAVI) data. Hereby, we report the feasibility of full and simplified LVDD assessment in severe COVID‐19 patients admitted to ICU, the incidence of LVDD, and its association with mortality.

## 2 METHODS

This study was conducted in parallel to the international ECHO‐COVID,^17^ a longitudinal observational study involving 14 ICU of tertiary teaching hospitals in eight countries and registered in www.clinicaltrials.gov (NCT 04414410). In the present study, we included patients with confirmed SARS‐CoV‐2 infection admitted to the ICU at *Policlinico‐San Marco University Hospital*, Catania, and receiving at least one critical care echocardiography (CCE) exam within the first 3 days of ICU admission or after escalation from non‐invasive to invasive respiratory support (while already admitted to ICU). We performed transthoracic echocardiography (TTE) with the aid of a portable machine *General Electric (GE) Venue Go R2* equipped with TDI software. An ICU physician with certified experience in CCE (FS) conducted all the exams. At the time of conducting the CCE, the operator was not blinded to therapies and the patient's history. Echocardiography calculations and interpretation of the data were performed off‐line (FS), with data checked by two other authors (LLV, VD). Data were collected in three major domains as suggested by the PRICES appraisal and statement^26^, ^27^: (a) patient characteristics (including co‐morbidities), (b) clinical data at the time of CCE exam (including hemodynamic and ventilation variables), and (c) echocardiography data. Data integrity and quality were examined thoroughly by a methodologist (SH).

### 2.1 Outcomes

Our primary outcomes were the feasibility of assessment of LVDD according to the latest ASE/EACVI 2016 recommendations^20^ and the incidence of LVDD according to these recommendations. In the current ASE/EACVI 2016 recommendations,^20^ two TDI data (e’ wave velocity and E/e’ ratio) are combined with tricuspid regurgitation jet velocity (TRvel) and LAVI for diagnosing LVDD. For patients with established LVDD diagnosis (including those with depressed LV ejection fraction, LVEF), the values of E wave and E/A ratio are then considered in the algorithm for LVDD grading into grades I, II, and III. Considering the challenges in the application of these recommendations to the ICU setting in mechanically ventilated patients,^28^, ^29^ we also conducted an analysis on the assessment of LVDD according to the protocol suggested by Lanspa et al., which combines abnormal values of septal e’ (for the diagnosis of LVDD) with values of E/e’ ratio for the grading of LVDD itself.^30^ As secondary outcomes we investigated the association between hospital mortality and the diagnosis of LVDD or each single echocardiographic variable collected for the study. A sensitivity analysis was conducted to evaluate the ICU mortality.

### 2.2 Data analysis

Demographics and clinical variables are reported with descriptive analysis in the overall population and according to the outcome of hospital mortality. In particular, categorical data were reported as numbers and percentages; median with interquartile range [IQR] is used to report continuous data as most of them were not normally distributed according to Kolgomorov‐Smirnoff test and/or Q–Q plot inspection. Missing values were not included in the analyses. Data imputation was not carried out due to the presence of data missing not at random.

Categorical variables were analyzed using Fisher's exact test. Continuous variables were analyzed with Mann‐Whitney tests. Due to the small sample size, no multivariate analysis or regression was conducted to correct for confounders. All statistical tests were two‐sided and were performed using the SPSS software (*IBM SPSS Statistics for Windows, Version 25.0. Armonk, NY: IBM Corp*.).

## 3 RESULTS

In total, 102 patients were admitted to our ICU during the study period, while our unit served as a general “clean” ICU during the other periods of the current pandemic. At our center, 35 patients (34% of those admitted) received advanced CCE within the first 3 days of ICU admission or after escalation from non‐invasive to invasive respiratory support while already admitted to ICU.

### 3.1 Study population and feasibility of LVDD assessment

Assessment of LVDD according to current ASE/EACVI guidelines^20^ was feasible in 26/35 patients (74%), and these patients were included in the study. Table 1 shows characteristics in the study population, both overall and according to hospital mortality (survivors, *n* = 9; non‐survivors, *n* = 17). In particular, we report baseline characteristics, comorbidities, and outcome data on length of stay and mortality, all together with ventilation support, arterial blood gas analysis and hemodynamic conditions at the time of advanced CCE. Non‐survivors had higher incidence of intubation and mechanical ventilation as compared to survivors (*p* = .03), and a trend toward a worse P/F ratio (*p* = .052).

**TABLE 1:** Characteristics of patients with coronavirus disease admitted to our intensive care unit (ICU) and receiving advanced critical care echocardiography examination

*Population*	*Overall* *n = 26*	*Survivors* *n = 9*	*Non‐survivors* *n = 17*	*p‐value*
*Baseline characteristics and comorbidities*				
Age (years)	71 [15.5]	66 [22]	72 [10]	.22
Weight (Kg)	76.5 [21.25]	80 [29]	75 [21]	.18
Height (cm)	170 [15]	170 [14]	170 [17]	.83
BMI (kg/m^2^)	27.0 [7.45]	27.7 [7]	25.4 [5.3]	.09
Smoking history (Y/N/Ex)	8 + 14 + 4	3 + 3 + 3	5 + 11 + 1	All ns
Cardiomyopathy ‐ HF	4 (15%)	0	4 (24%)	.26
Hypertension	16 (62%)	4 (44%)	12 (71%)	.23
Pacemaker	0	0	0	–
Pulmonary disease ‐ COPD	6 (23%)	1 (11%)	5 (29%)	.38
Diabetes mellitus	1 (4%)	0	1 (6%)	1.00
Chronic kidney disease	4 (15%)	1 (11%)	3 (18%)	1.00
Beta‐blockers	7 (27%)	2 (22%)	5 (29%)	1.00
ACE‐inhibitor ‐ ARB	7 (27%)	1 (11%)	6 (35%)	.36
*Ventilation data at the time of echocardiography*				
Ventilation mode				**.0 3**
ETI (pressure control mode)	17 (65%)	3 (33%)	14 (82%)	(ETI vs. others)
NIV + HFNC	7 + 2 (35%)	5 + 1 (67%)	2 + 1 (18%)	
FiO_2_ (%)	70 [25]	60 [29]	75 [33]	.18
Respiratory rate (bpm)	18 [7.25]	21 [7]	18 [7]	.22
Tidal volume (ml, if ETI)	485 [55]	490	480 [70]	.70
PEEP (cmH_2_O)	10 [4]	10 [4]	10 [3]	.87
P/F ratio	105 [65.7]	142 [77.5]	99 [44.3]	.052
SaO_2_ (%)	96 [4.25]	97 [3]	96 [5]	.31
*Arterial blood gas data at the time of echocardiography*				
PaO_2_ (mmHg)	78 [24.2]	81 [27]	71.50 [26]	.36
PaCO_2_ (mmHg)	42 [15.5]	42 [14]	42 [26]	.63
pH	7.41 [.09]	7.44 [.11]	7.40 [.08]	.09
Base deficit (mmol/L)	1.40 [4.9]	2.2 [6.0]	.6 [6.7]	.08
Lactate (mmol/L)	1.8 [1.0]	1.5 [.7]	1.8 [1.9]	.49
Time to CCE (days)	1 [1.25]	2 [3]	1 [1]	.83
*Hemodynamic data at the time of echocardiography*				
Heart rate (bpm)	71 [25]	71 [16]	71 [39]	.96
Atrial fibrillation (*n*)	4 (15%)	0	4 (24%)	.26
Systolic arterial pressure (mmHg)	119 [31.5]	129 [38]	118 [26]	.92
Diastolic arterial pressure (mmHg)	64 [19]	65 [19]	64 [18]	.49
Mean arterial pressure (mmHg)	84 [21.5]	92 [23]	84 [24]	.49
NE infusion (*n*)	6 (23%)	1 (11%)	5 (29%)	.38
Dose of NE (if used, μ/kg/min)	.04	‐	‐	
*Outcome data*				
ICU length of stay	15 [9.75]	18 [22]	11 [9]	**.0 1**
ICU mortality	15 (58%)	–	–	–
Hospital mortality	17 (65%)	–	–	–

*Notes*: We present results in different sub‐sections: (a) baseline characteristics and comorbidities; (b, c, and d) ventilation, arterial blood gas analysis, and hemodynamic conditions at the time of echocardiography; (e) outcome data on length of stay and mortality. Data are reported in the overall population and according to hospital mortality, and are expressed as median [interquartile range] or as number and/or percentage. P values below 0.05 are indicated in bold and underlined.

Abbreviations: ACE, angiotensin converting enzyme; ARB, angiotensin receptor blockers; BMI: body mass index; COPD, chronic obstructive pulmonary disease; ETI, endotracheal intubation; HF, heart failure; NE, norepinephrine; NIV, non‐Invasive Ventilation; PEEP, positive end expiratory pressure.

### 3.2 Echocardiographic data and incidence of LVDD

Table 2 reports the CCE data in study population. These data are grouped according to LV size, systolic, and diastolic function, along with data on RV size and systolic function, inferior vena cava size (IVC) and pericardium. According to the ASE/EACVI 2016 guidelines,^20^ 12 patients had LVDD (46%). When performing the grading, we found one patient with LVDD grade I, four patients with grade II, and seven with indeterminate grade (could be I or II).

**TABLE 2:** Echocardiography data of patients with coronavirus disease admitted to our intensive care unit (ICU) and receiving advanced critical care echocardiography examination

Population		Overall *n* = 26	Survivors *n* = 9	Non‐survivors *n* = 17	*p*‐value
LV size and systolic function					
LV size (visual)					.43 (normal vs. others)
Normal size		14 (54%)	6 (67%)	8 (47%)	
Reduced size		8 (31%)	3 (33%)	5 (29%)	
Moderate‐severe dilatation		4 (15%)	0	4 (24%)	
LVEDD (mm)		38.5 [14.75]	34 [11.5]	39 [17]	.24
LVEDV (ml)		63.5 [58.25]	60 [48]	66 [71]	.46
LVEF (%)		60 [12.5]	61 [10]	55 [25]	.49
s’ (cm/sec)	Average	6.7 [4.25]	10 [4.5]	6.1 [2.4]	**.0 1**
	Septal	6.9 [3.5]	10 [4]	6.1 [3.3]	**.0 3**
	Lateral	7 [4.5]	9 [5]	5.7 [3.8]	**.0 2**
LV systolic function (visual)					.09 (normal vs. others)
Normal		16 (62%)	8 (89%)	8 (47%)	
Hyperkinetic		6 (23%)	1 (11%)	5 (29%)	
Moderate‐severe hypokinetic^*^		4 (15%)	0	4 (24%)	
LV diastolic function					
ESC guidelines					.11
Normal		14 (54%)	7 (78%)	7 (41%)	
Abnormal		12 (46%)	2 (22%)	10 (59%)	
[Grade I/II/III/indet. ‐ *n*]		[1‐4‐0‐7]	[0‐0‐0‐2]	[1‐4‐0‐5]	
Lanspa definition					1.00
Normal (e’ septal > 8)		5 (19%)	2 (22%)	3 (18%)	
Abnormal (e’ septal < 8)		21 (81%)	7 (78%)	14 (82%)	
[Grade I/II/III (E/e’) ‐ *n*]		[3‐7‐11]	[2‐2‐3]	[1‐5‐8]	
LAVI (AL‐method, ml/m^2^)		33.9 [40.7]	28.8 [17.3]	37.2 [59.5]	.49
LAVI_(MODs, ml/m^2^)		29.9 [39.7]	27.2 [15.2]	31.1 [53]	.53
TR jet velocity (cm/sec ‐ *n* = 12)		2.7 [.8]	2.6 [1.1]	2.7 [.9]	1
e’ (cm/sec)	Average	6.7 [2.4]	7 [3]	6 [3]	.29
	Septal	5 [3]	7 [3]	5 [2]	.31
	Lateral	7 [3]	8 [3.5]	7 [4.6]	.30
E/e’ ratio	Average	10.8 [4.8]	8.2 [4.5]	11.0 [5.1]	.09
	Septal	12.1 [5.3]	9.75 [6.5]	12.60 [7]	.22
	Lateral	9.7 [4.8]	7.4 [3.6]	10.5 [6.3]	**.0 3**
E wave (cm/sec)		67.5 [22.12]	69 [24]	66 [24]	.60
A wave (cm/sec)		75.7 [23.8]	75.4 [30]	75.9 [18]	.70
E/A ratio		.82 [.41]	.80 [.4]	.87 [.4]	.74
Deceleration time (msec)		252.5 [102.7]	325 [135]	241 [79]	**.0 1**
RV size and systolic function					
RV systolic function					.69 (normal vs. others)
Normal		16 (62%)	5 (56%)	11 (65%)	
Hyperkinetic		7 (26%)	3 (33%)	4 (23%)	
Hypokinetic*		3 (12%)	1 (11%)	2 (12%)	
TAPSE (mm)		20 [7.5]	20 [12]	20 [8]	.75
RV/LVEDA ratio		.45 [.11]	.45 [.2]	.40 [.2]	.29

*Notes*: Variables are reported according to left ventricular (LV) size, systolic and diastolic function, right ventricular (RV) size and systolic function, inferior vena cava size (IVC), pericardium, and valves.

Data are reported both in the overall population and according to hospital mortality. Evaluation of LV diastolic dysfunction is performed according to two approaches: the European Society of Cardiology (ESC) recommendations and the protocol suggested by Lanspa et. al. As most of the data are not normally distributed, continuous variables are reported as median and [interquartile range]. Categorical variables are reported as numbers and/or percentages. P values below 0.05 are indicated in bold and underlined.

Abbreviations: A‐L, area‐length method; LAVI, left atrial volume index; LVEDA, LV end‐diastolic area; LVEDD, LV end‐diastolic diameter; LVEDV, LV end‐diastolic volume; LVEF, LV ejection fraction; MODs, method of disks TAPSE, tricuspid annular plane systolic excursion; TR, tricuspid regurgitation.

Diagnosis of LVDD according to the simplified Lanspa criteria^30^ was made in 21 patients (81%). Of note, over half of these patients diagnosed with LVDD according had grade III dysfunction (*n* = 11/21), followed by grade II (*n* = 7) and grade I (*n* = 3).

**FIGURE 1:**
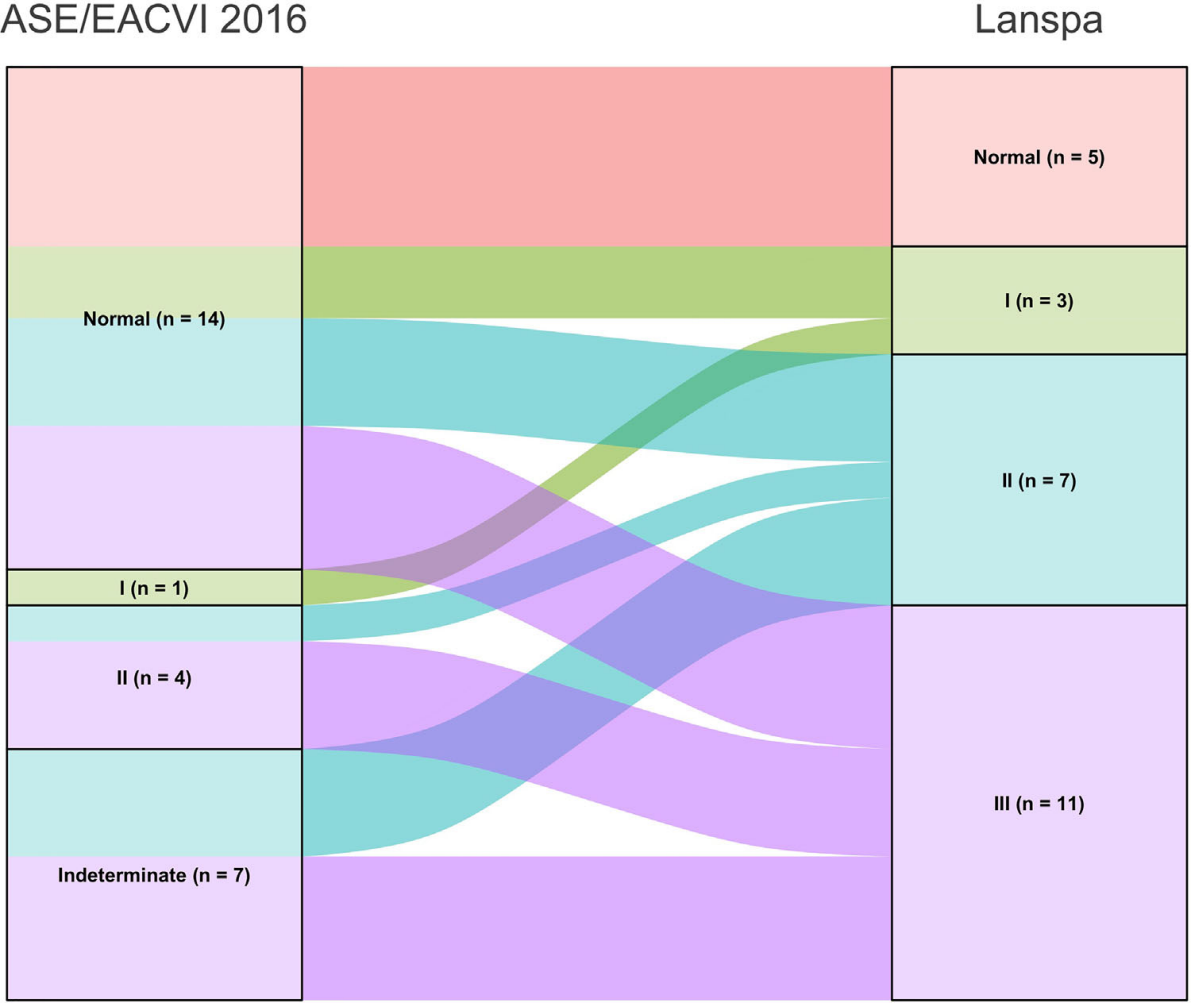
Alluvial Plot describing differences in diagnosis and grading of left ventricular diastolic dysfunction according to the two definitions. ASE, American Society of Echocardiography; EACVI, European Association of Cardiovascular Imaging.

**FIGURE 2:**
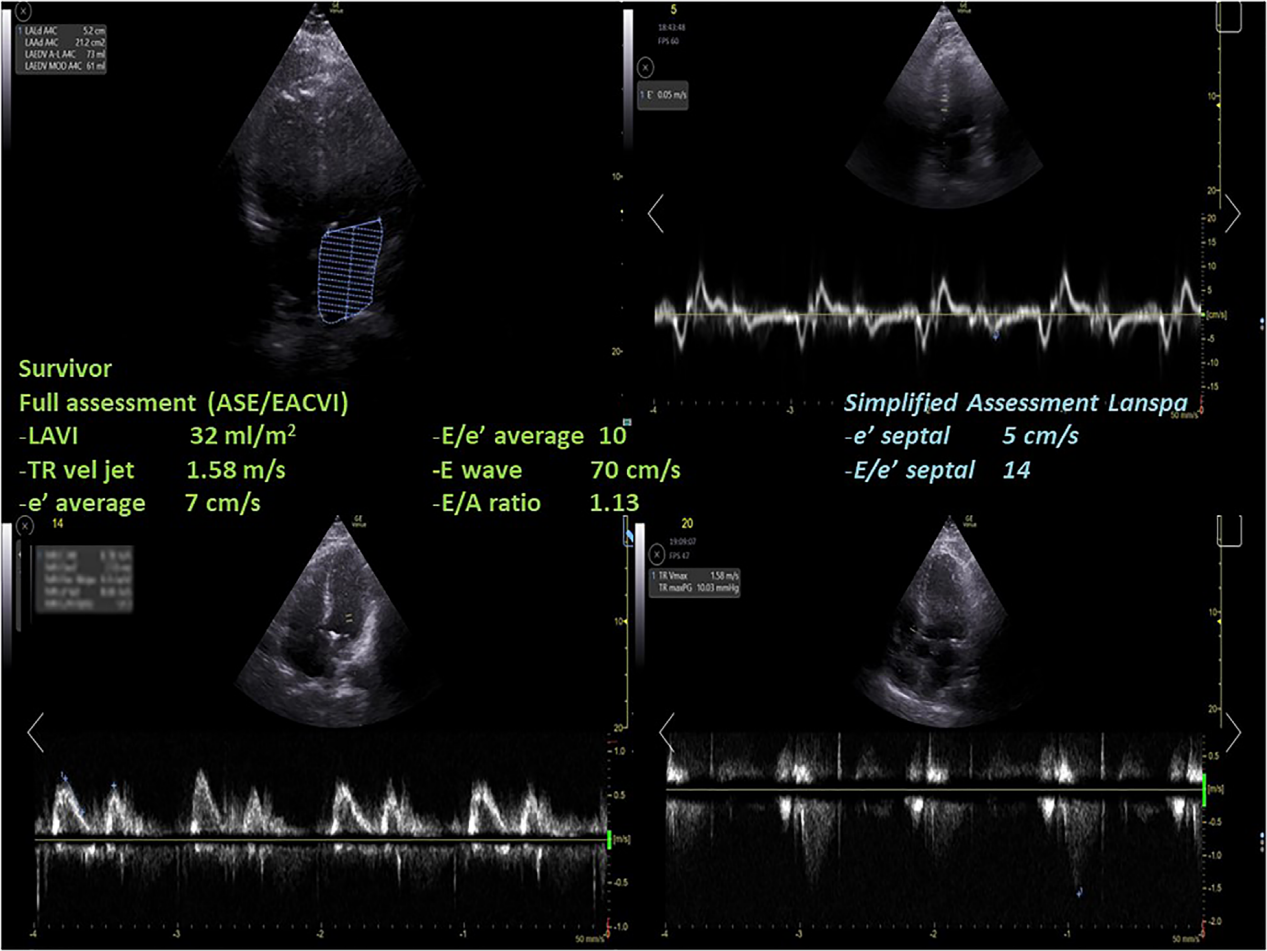
A case of a survivor where the assessment with full criteria resulted in a normal left ventricular diastolic function, while using the simplified assessment according to Lanspa criteria a diagnosis of grade III left ventricular diastolic dysfunction was made. ASE, American Society of Echocardiography; EACVI, European Association of Cardiovascular Imaging; LAVI, left atrial volume index; TR, tricuspid regurgitation.

**FIGURE 3:**
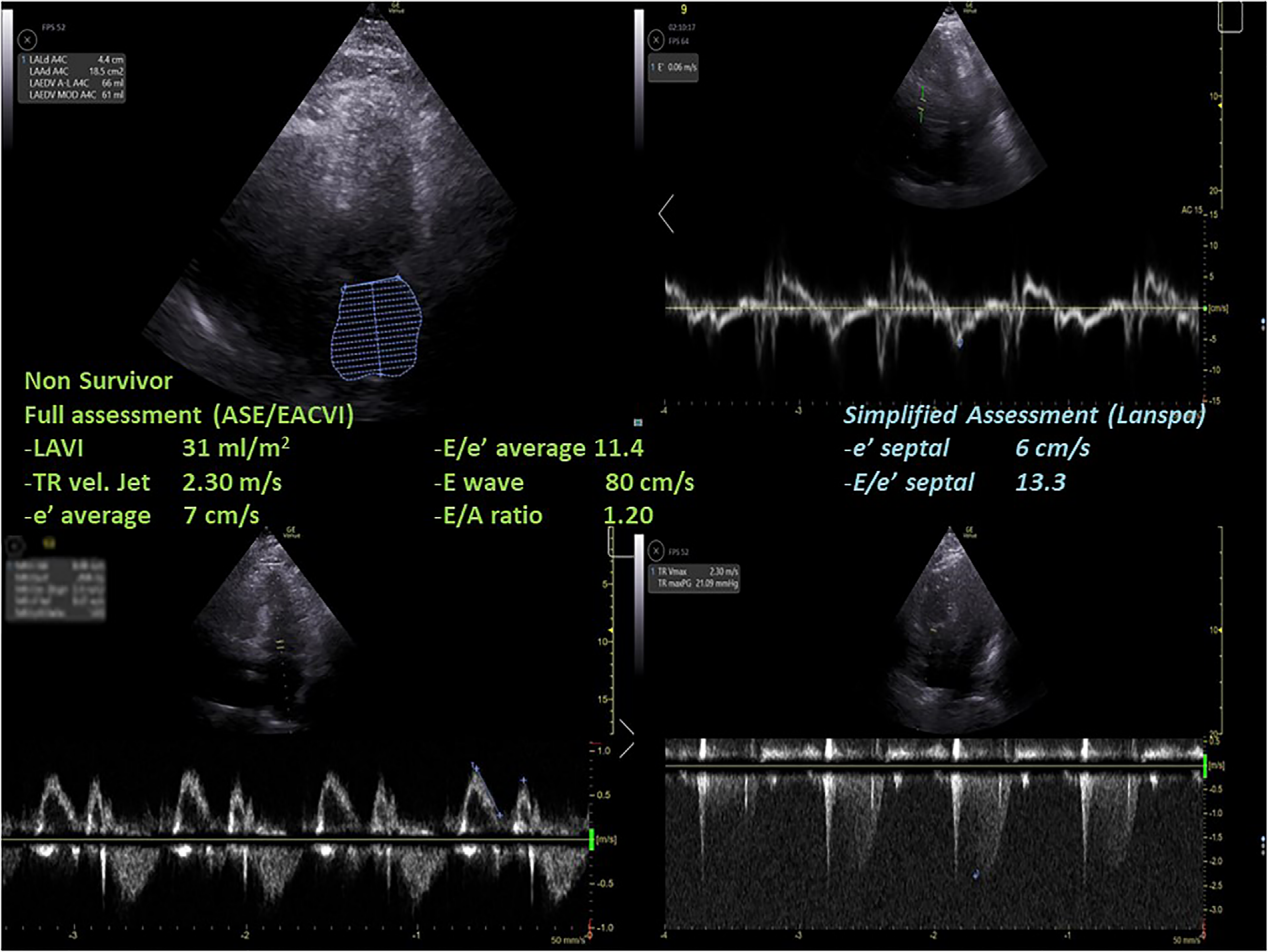
A case of a non‐survivor where the assessment with full criteria resulted in a normal left ventricular diastolic function, while using the simplified assessment according to Lanspa criteria a diagnosis of grade III left ventricular diastolic dysfunction was made. ASE, American Society of Echocardiography; EACVI, European Association of Cardiovascular Imaging; LAVI, left atrial volume index; TR, tricuspid regurgitation.

Differences in LVDD diagnosis and in grading according to the ASE/EACVI 2016^20^ and the simplified Lanspa criteria^30^ are shown in an alluvial plot (Figure 1). Two examples of pronounced differences in the assessment (the full one resulting in normal diastolic function, and the simplified one describing a grade III LVDD) are reported in Figure 2 (survivor) and Figure 3 (non‐survivor).

### 3.3 Diagnosis of LVDD and hospital mortality

We found no differences in hospital mortality according to the diagnosis of LVDD based on the ASE/EACVI 2016 recommendations. Among non‐survivors, *n* = 10/17 (59%) had a diagnosis of LVDD as compared with survivors (*n* = 2/9, 22%; *p* = .11). The sensitivity analysis performed according to ICU mortality held similar results (*n* = 9/15 vs. *n* = 3/11, respectively; *p* = .13).

When investigating association between hospital mortality and LVDD diagnosis according to simplified Lanspa criteria,^30^ *n* = 14/17 (82%) of hospital non‐survivors and *n* = 7/9 (78%) of survivors had a diagnosis of LVDD (*p* = 1.00). We also found no differences in the diagnosis of LVDD according to ICU mortality (*p* = .50).

Numbers were far too small to analyze subgroups according to LVDD grade, and these analyses were not conducted.

### 3.4 Single echocardiographic parameters and association with hospital mortality

Regarding the secondary outcomes focusing on the association between single echocardiographic parameters and hospital mortality, we found that non‐survivors had higher lateral E/e’ ratio (*p* = .03). A trend toward higher average E/e’ ratio was also found (*p* = .08), while septal values were not significantly different (*p* = .31). Deceleration Time, a parameter used in previous guidelines (ASE/EACVI 2009) for the assessment of LVDD,^31^ was significantly different according to hospital survival (*p* = .01). The only other echocardiographic parameter significantly associated with hospital survival was the TDI s’ wave. This result was consistent with all measurements performed as septal, lateral, and average (*p* = .02; *p* = .02; and *p* = .01, respectively).

PRICES Checklist for reporting echocardiography studies are provided as Supplementary Digital Contents (1, for the checklist of the common items; 2, for checklist of the echocardiography variables studied). All the essential items of the LV diastolic function domain were reported, together with several items of the LV systolic function and RV function ones.

## 4 DISCUSSION

In this small single center study conducted in patients with COVID‐19 admitted to ICU, full assessment of LV diastolic function according to the ASE/EACVI 2016 guidelines^20^ was feasible in roughly three quarter of the population. The challenge of performing this assessment in the remaining patients is not entirely surprising as the assessment requires a good apical four‐chamber acoustic window with proper alignment, recording, and calculation of several parameters. Several factors may contribute to the challenges of properly assessing LVDD in COVID‐19 patients. First, considering the severe respiratory impairment of this population of patients and the use of high positive end‐expiratory pressures (median 10 cmH_2_O in our study), it is not unusual to experience suboptimal acoustic windows. Second, performing advanced CCE under hazardous conditions wearing personal protective equipment and double gloves may be challenging, especially during a period of unprecedented clinical workload; in such cases, advanced assessment of LVDD may be perceived as cumbersome and time‐consuming, and it is unlikely to become a priority in a busy and understaffed ICU. Further, severe COVID‐19 patients are frequently treated with prone position, which may render more complex the assessment with CCE.^32^, ^33^

With several limitations, this study is probably one of the few available experiences reporting full LVDD assessment according to the current ASE/EACVI 2016 guidelines.^20^ Indeed, while several studies reported behavior of one or more echocardiography variables used for the assessment of LVDD, it seems no studies have reported full LVDD assessment according to the latest guidelines,^20^ as shown by a systematic review.^9^ From an overview of the literature on COVID‐19 patients, we also could not find any experiences comparing the full and the simplified assessment of LVDD.

Unfortunately, our study is severely underpowered for detecting the influence of LVDD on the outcome of severe COVID‐19 patients. This was behind our control as the ICU served as COVID‐ICU for the Trust only for a brief period of time (∼4 months, *n* = 102 COVID‐19 admissions); moreover, the workload did not always allow timely assessment with advanced CCE for the purpose of this study, as only one operator had advanced CCE skills and joined the ECHO‐COVID study. Therefore, all together with the risk of statistical error, it is likely that an inevitable selection bias took place.

We found that almost half of COVID‐19 patients were diagnosed with LVDD according to ASE/EACVI 2016 guidelines^20^ (*n* = 12/26, 46%). LVDD was associated with a trend toward higher mortality in those with LVDD according to ASE/EACVI 2016 guidelines^20^ (hospital, *p* = .11; ICU, *p* = .13). Conversely, the assessment of LVDD according to simplified Lanspa criteria^30^ showed no statistical differences; of note, LVDD diagnosis with the latter criteria was made in over 80% of patients (*n* = 21/26), demonstrating significant differences with the assessment according to ASE/EACVI 2016 guidelines.^20^ For educational purposes we illustrate two of our cases (one survivor and one non‐survivor; Figures 2 and 3, respectively) where assessment with full criteria resulted in a normal LV diastolic function, while using the simplified assessment both patients were classified as grade III LVDD . The reason of this striking difference relies probably in the large number of patients with depressed TDI e’ wave values in the overall population; indeed, depressed e’ velocity is the only criteria adopted by Lanspa et al.^30^ for the diagnosis of LVDD. Moreover, applying the simplified Lanspa criteria for LVDD grading^30^ (based on values of E/e’ ratio) over half of patients had grade III LVDD (*n* = 11/21) followed by grade II (*n* = 7) and grade I (*n* = 3). Taken together, these results show huge differences in the assessment of LVDD and probably the use of the simplified criteria for diagnosis and grading of LVDD in patients with severe COVID‐19 should be considered cautiously as likely to produce some degree of overestimation. For instance, half of the patients diagnosed with normal LV diastolic function according to the ASE/EACVI 2016,^20^ had grade II (*n* = 3) or III (*n* = 4) LVDD according to the simplified definition.^30^

Bearing in mind the limitations of the study, we think that our analysis is in line with previous experience reporting the importance of LVDD in the context of critical illness and also with the known difficulties in assessing LVDD in mechanically ventilated patients.^34^ Different phenotypes of cardiovascular dysfunction have been described in critically ill patients,^35^ and LVDD has received attention for its association both with mortality in septic patients^21^, ^22^ and for weaning failure.^23^ Conversely, LVSD has not shown the same association when evaluated by means of LVEF^36^ or s’ wave^24^ in critically ill patients. It was somewhat unexpected to find that TDI s’ wave was significantly lower in hospital (and ICU) non‐survivors, as this parameter has not been found associated with prognosis in critically ill patients (i.e., septic patients^24^); moreover, the population we studied was mostly free from cardiovascular support (77%), and those on norepinephrine received a very low dose (.04 mcg/kg/min). However, considering that a myocarditis‐like pattern has been found in cardiac magnetic resonance imaging after COVID‐19 also in cohorts of asymptomatic and mildly symptomatic patients,^12^, ^37^, ^38^ it is possible that the lower TDI s’ values are related to an impaired longitudinal LV systolic function not detected by assessment of LVEF.

We also found that lateral E/e’ ratio was significantly higher in non‐survivors at hospital discharge, followed by a trend in average E/e’ ratio (*p* = .08). The mean difference between survivors and non‐survivors was just over 3 points, opening the possibility that higher left atrial pressure contributes to poorer prognosis in patients with severe COVID‐19. However, the overall values of E/e’ ratio were not very high (median value of average E/e’ was 10.8), and non‐survivors presented median values of 11, well‐below the cut‐off suggested by the ASE/EACVI 2016 guidelines (E/e’ 14).^20^ From clinical perspectives, this finding is in line with lung edema and impaired gas exchange mainly triggered by interstitial pneumonia, with left atrial pressure playing a marginal role in these cases. In our opinion, it is reasonable that E/e’ ratio does not play a major role also in consideration of the gradual course of the COVID‐19 disease. Indeed, in most of the cases evolving toward severe interstitial patterns, the progression happens over days or weeks. During this period, the patient has already experienced fever and dehydration. The admission to the Emergency Department or to other COVID‐19 areas with prolonged oxygen support (high‐flow or non‐invasive ventilation) increases the likelihood of intravascular volume depletion due to sweating (fever) and poor water intake. In such cases, the presence of normal left atrial pressure may be related to a reduced circulating volume for the above‐described reasons, and it does not necessarily reflect intrinsic myocardial relaxation. On the contrary, the TRvel (other parameter used for assessing LVDD) could increase during severe COVID‐19 due to the occurrence of micro‐ or macro‐vascular thrombosis/embolism in the pulmonary circulation or for the effects of mechanical ventilation, rather than as a reflection of an ongoing impaired LV relaxation (post‐capillary). Therefore, there are several adjunctive differences and peculiarities that may render the evaluation of LVDD even more complex as compared to the usual ICU patient. Among these, COVID‐19 usually has a more gradual evolution of the critical illness as compared to typical septic shock evolving more rapidly.

### 4.1 Limitations

We already mentioned the small sample size and the non‐consecutive enrollment as main limitation of this ancillary study. In consideration of the small sample size, we thought that performing sophisticated multivariate and/or regression statistical analyses with the aim to address for confounders would have not been meaningful. Although we reported the items for the study interpretation according to the PRICES checklist, this does not rule out at all the interference of these confounders on our results. Another consideration is about the implementation of vaccination worldwide and the presence of new variants. These factors have largely influenced the circulation and the clinical course of the COVID‐19 with a reduction in severe cases and drop in ICU admission. These factors should be accounted when comparing our results with future studies, as a different degree of cardiovascular impairment with new variants or as result of vaccination cannot be excluded.

## 5 CONCLUSIONS

In a small single‐center study, the assessment of LVDD according to latest ASE/EACVI 2016 guidelines was feasible in three quarter of COVID‐19 patients admitted to ICU. Assessment with a simplified definition based on TDI values only yielded very different results. Hospital non‐survivors showed a non‐significant trend toward greater LVDD incidence with full assessment but not with simplified diagnostic criteria. Non‐survivors had significantly worse s’ values (all) and higher E/e’ ratio (lateral).

## CONFLICT OF INTEREST

The authors declare no conflict of interest.

## Supporting information




Supporting Information
