# Supplementary material for: Full and simplified assessment of left ventricular diastolic function in covid‐19 patients admitted to ICU: Feasibility, incidence, and association with mortality
Source: Echocardiography. 2022 Oct 6;39(11):1391–400. doi: 10.1111/echo.15462 (PMC9827986; doi:10.1111/echo.15462)
Supplement: Supplementary file 1 — Supporting Information [file ECHO-39--s001.docx]

**SUPPLEMENTAL DIGITAL CONTENT**

1. **SDC 1. Prices Checklist Common Items**

**
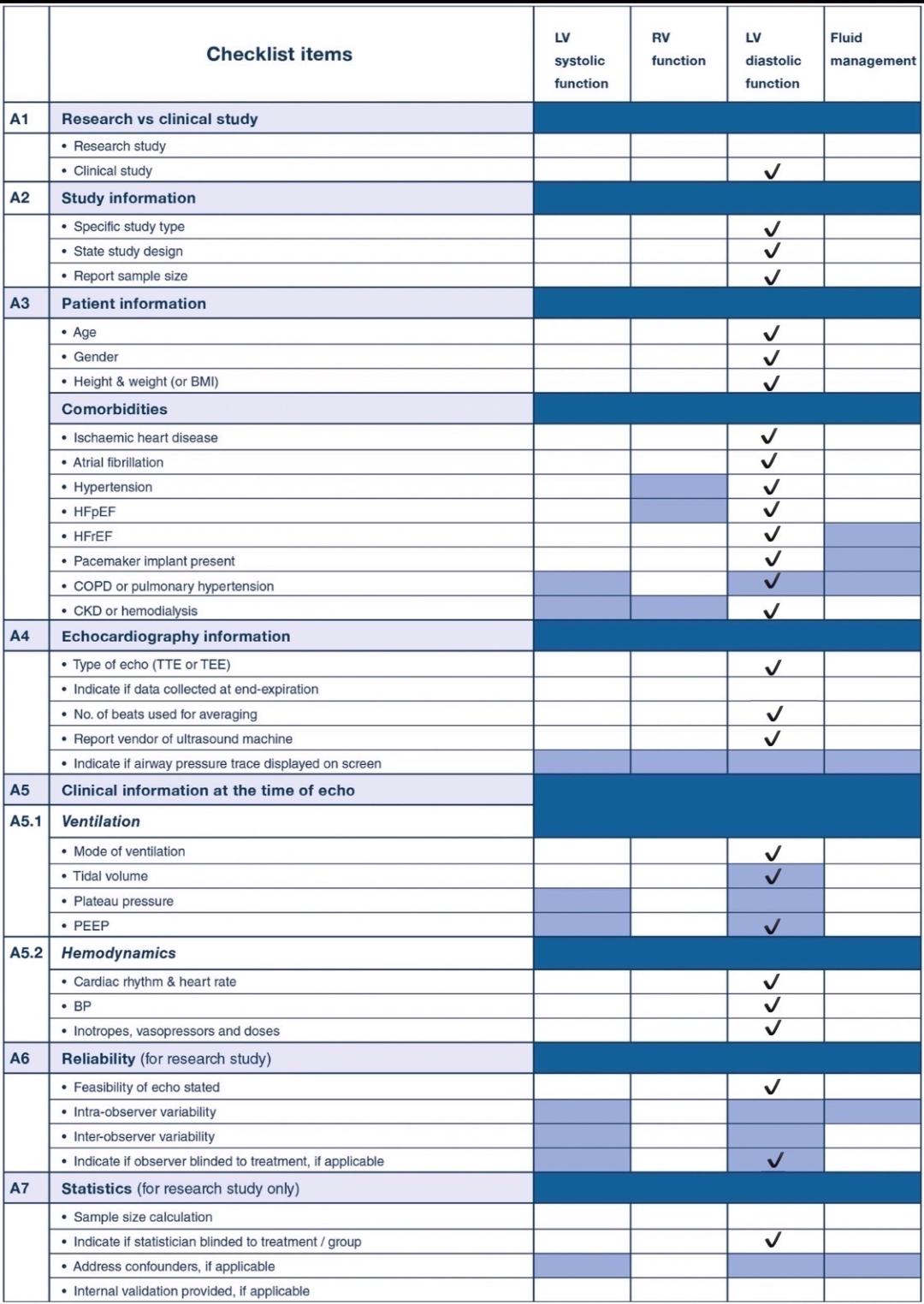
**

1. **SDC 1. Prices Checklist Specific Items**

**
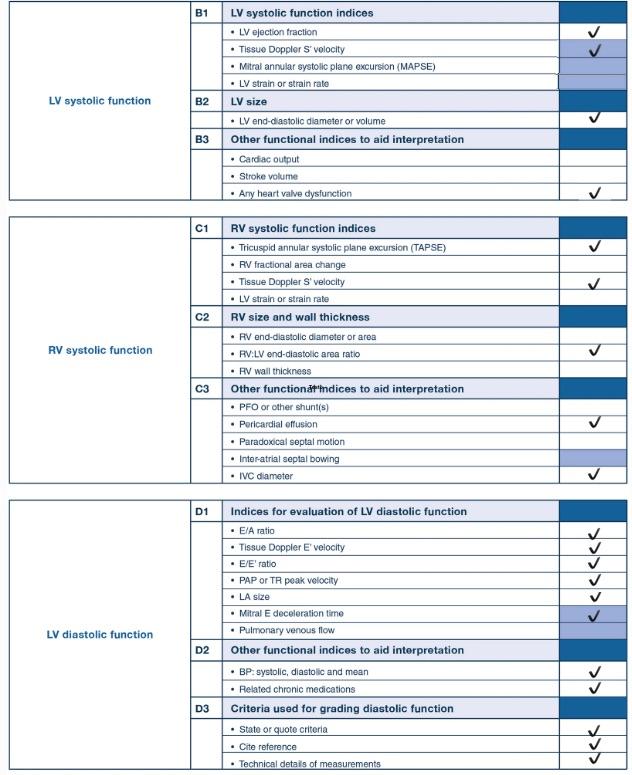
**
